# Experimental research in rats on the reactivity of new corneal blood vessels to adrenaline

**DOI:** 10.22336/rjo.2021.12

**Published:** 2021

**Authors:** Daniela Bianca Damian, Aurelian Mihai Ghiță, Sânziana Istrate, Ioana Cristina Coman

**Affiliations:** * Department of Ophthalmology, “Dr. Alexandru Popescu” Military Emergency Hospital Focșani, Focșani, Vrancea, Romania; ** Department of Physiology, “Carol Davila” University of Medicine and Pharmacy, Bucharest, Romania; *** Department of Ophthalmology, “Carol Davila” University of Medicine and Pharmacy, Bucharest, Romania; **** Department of Ophthalmology, “Carol Davila” University of Medicine and Pharmacy, Bucharest, Romania

**Keywords:** adrenaline, new blood vessels, cornea, vasoconstriction

## Abstract

**Aim:** The purpose of this experimental study was to evaluate the existence of adrenergic receptors in ketamine-induced corneal blood vessels in rat pups.

**Methods:** The study of corneal neovascularization motricity was performed on 45-day-old Wistar rats in which, starting from the 15th day of life, corneal blood vessels were obtained by injecting intraperitoneal ketamine at a dose of 150 mg/ kg body weight, a total of 5 successive doses. The examination of the neovascularization was done with the help of a Nikon stereomicroscope connected to a video camera and a computer, the total magnification being 400X. The reactivity of the new corneal blood vessels to the administration in conjunctival instillations of a 1.5 mmol/L adrenaline solution was tested. The parameters followed were represented by variations in the caliber of corneal blood vessels. The data were analyzed using Microsoft Office Excel.

**Results:** Administration of distilled water did not produce statistically significant changes in corneal blood vessels, while adrenaline produced a statistically significant constriction of vascular diameter (p=0.01 at T9, p=0.004 at T10, p=0.019 at time T11 of examinations).

**Conclusions:** The results showed that adrenaline produces vasoconstriction in the new corneal blood vessels, which allows us to assume that they contain α-adrenergic receptors. However, we cannot say that corneal pathological vessels do not contain β2-type adrenergic receptors, because the effect of adrenaline may be an algebraic sum between vasoconstriction produced by stimulating α-adrenergic receptors and vasodilation produced by stimulating β2-adrenergic receptors, but in which the vasodilating effect may be masked by the vasoconstrictor effect given by a higher density of α-adrenergic receptors.

**Abbreviations:** A= adrenaline, DNM = non-measurable diameter, NA= noradrenaline, Std.Er.= Standard error

## Introduction

The adrenergic system comprises all the structures that use the catecholamines adrenaline and noradrenaline as chemical mediators. First described by Ahlquist in 1948 [**1**], adrenergic receptors are divided into two categories: α-adrenergic receptors and β-adrenergic receptors. Subsequently, the sub-types α1 with postsynaptic localization and α2 located predominantly presynaptic, but also postsynaptic and extrasynaptic were highlighted for α-adrenergic receptors [**2**-**4**]. α-adrenergic receptors were also divided into several sub-types β1, β2, β3, and β4 [**5**-**7**]. In 1959, Furchgott discovered other types of adrenergic receptors, gamma, and delta [**8**], responsible for the actions of catecholamines in smooth muscles. Currently, most authors admitted the existence of α1-, α2-, β1- and β2-adrenergic receptors. α1-adrenergic receptors are found mainly in the vascular smooth muscles, and their stimulation causes vasoconstriction. α2-adrenergic receptors are located mainly presynaptically, and their stimulation inhibits presynaptic release of norepinephrine leading to relaxation of vascular and intestinal smooth muscles. There are also postsynaptic α2-receptors that cause vasoconstriction [**9**]. Stimulation of β2-adrenergic receptors causes vasodilation by relaxing the vascular smooth muscles (arteriolar and venous).

The corneal epithelium expresses α- and β-type adrenergic receptors. β-adrenergic receptors are found at the cell surface, and through adenylate cyclase, leading to the formation of AMP-cyclic, with stimulation of Cl- permeability at the epithelial membrane [**10**], as well as α1-adrenergic receptors that regulate inositol-phosphate turnover [**11**]. Stimulation of corneal β-adrenergic receptors causes protein kinase A activation and an increase in intracellular cAMP concentration, and stimulation of α2-adrenergic receptors inhibits protein kinase A (PKA) activity by inhibiting adenylate cyclase. Modulation of the corneal cAMP-PKA pathway can play important roles in homeostasis and corneal wound healing [**12**].

The effects of adrenaline and noradrenaline depend on their selectivity to the types of adrenergic receptors, as well as the density of the types of adrenergic receptors in the tissues. Noradrenaline has a high affinity for α-adrenoreceptors, causing a pressor-type response. Noradrenaline causes vasoconstriction in all vascular territories and increased volume by contraction of the spleen capsule [**9**]. Adrenaline has an affinity for both types of receptors, thus determining biphasic actions, the final response depending on the types of receptors it binds to and their density at the cell surface. Thus, in the cutaneous, mucosal, and splanchnic territories, where there is a higher density of α1-adrenergic receptors, adrenaline leads to vasoconstriction, while in the brain, in striated muscles, kidneys, or coronary adrenaline causes vasodilation due to an increased density of β2-adrenergic receptors. **Table 1** shows a distribution of adrenergic receptors in the ocular tissues (in humans), and **Table 2** shows a classification of adrenoceptors with agonists (endogenous and exogenous) and antagonists.

**Tabel 1:** Distribution of adrenergic receptors in human eye tissues

Type of adrenergic receptor	Tissue location
α1	- iris dilator muscle [**13**]; retinal blood vessels [**14**]; ciliary muscle [**15**]; conjunctival epithelium [**16**]; corneal epithelium [**11**] and endothelium [**17**]
α 2	- retinal pigmented epithelium- choriocapillaris, neurosensory retina [**18**]; iris epithelium and ciliary epithelium [**18**,**19**]; ciliary muscle [**19**]; retinal blood vessels [**14**]; retina (ganglion cells, and cells in the inner and outer nuclear layers) [**20**]; conjunctival epithelial cells [**16**,**21**]; trabecular meshwork cells [**22**]
β1	- iris-ciliary body (small number) [**23**]; conjunctival epithelium [**16**]; retinal blood vessels [**24**]
β 2	- ciliary muscle [**15**]; trabecular meshwork cells [**25**-**27**]; corneal epithelium and endothelium, lens epithelium, retina [**27**]; conjunctival epithelium [**16**,**27**]
β 3	- conjunctival epithelium [**16**]; retinal endothelial cells [**28**]

**Tabel 2:** Classification of adrenergic receptors with agonists (endogenous and exogenous) and antagonists

*Type of adrenergic receptor*	Agonists (endogenous)	Selective agonists (exogenous)	Selective antagonists
α1	NA=A [**29**,**30**]	-phenylephrine [**31**]	-prazosin [**33**]
		-methoxamine [**32**]	-doxazosin [**33**,**34**]
			-terazosin [**33**,**34**]
			-tamsulosin [**34**]
α2	A=NA [**30**,**31**]	-clonidine [**35**]	-yohimbine [**35**]
		-dexmedetomidine [**35**]	
		-oxymetazoline [**31**]	
β1	NA=A [**36**]	-dobutamine [**37**]	-metoprolol [**38**,**39**]
			-atenolol [**38**,**39**]
			-bisoprolol [**38**,**39**]
β2	A>NA [**36**]	-fenoterol [**40**]	-Butoxamine [**41**]
		-terbutaline [**40**]	
		-salbutamol [**40**]	
β3	NA>A [**36**]	-mirabegron [**42**]	-SR59230A [**43**]
NA = noradrenaline, A = adrenaline			

In certain situations, ocular neovascularization can lead to impaired visual function as well as the loss of the eyeball. The study of pharmacological receptors developed in the new blood vessels remains a challenge for the discovery of drug active substances, without/with minimal side effects, which may lead to their stopping or regression.

## Material and method

The experiments were performed on Wistar rats. The animals were provided by the Biobase of “Carol Davila” University of Medicine and Pharmacy, Bucharest. The batches of animals were brought to the working laboratory where they were kept in standard environmental conditions. The animals had ad libitum access to food and water and were housed in plexiglass cages. The ambient temperature was between 21 and 24 °C, and the relative humidity was maintained between 45 and 60%.

The experiment started with 75 rat pups, aged 15 days, in which corneal blood vessels were obtained by successive administration of ketamine at a dose of 150 mg/kg body weight, at an interval of 5 days between administrations, a total of five doses, to obtain a possible experimental model of corneal neovascularization. After the fifth dose of ketamine, rats with at least one eye neovascularization were selected, so that 19 batches of animals were formed, each batch of 6 eyes/experiment evaluable from the point of view of corneal neovascularization, on which adrenergic, cholinergic, and histaminergic substances were tested. The batch on which the adrenaline was tested was composed of 4 animals/6 eyes with neovascularization. The testing of the reactivity of new corneal blood vessels to adrenergic substances was performed on 45-day-old rats weighing 47-75 grams, in which corneal blood vessels were obtained by the method described above. Recordings were made for each eye with neovascularization, 6 eyes for each experiment.

The experiments were carried out with the approval of the Ethics Commission of “Carol Davila” University of Medicine and Pharmacy Bucharest, as well as following the provisions of Directive 2010/ 63/ EU on the protection of animals used for scientific purposes, as well as their transposition into national law, by Law No. 43/ 2014.

The substances used were ketamine 10% solution (CP-Ketamine 10%, CP-Pharma, Germany, veterinary medicine), distilled water (Zentiva SA, Romania), adrenaline Therapy 1 mg/ ml solution for injection adrenaline (SA Therapy, Romania).

Adrenaline and distilled water were administered as solutions in conjunctival instillations, and ketamine was administered by injection, intraperitoneally.

A Nikon stereomicroscope, model SMZ 1270, connected to a Mshot video camera, model MSX2-C, was used to visualize the corneal blood vessels, and the video camera was connected to a computer. The video camera was equipped with an intermediate lens attached to the front of the sensor to compensate for the magnification given by the stereomicroscope eyepieces. The system was manually calibrated using the “Mshot Imaging Analysis System” software and the Nikon micrometric calibration blade, type B (1 Div = 0.1 mm = 100 µm), J28004 series. The total magnification was 400X.

The anesthetized rats were placed in lateral decubitus in a restraint device to have optimal access to the eyeball to be examined, and the eyelid slit was kept open by manual traction. The examination was performed for each eye that developed corneal neovascularization. Image recording was performed at set time intervals of 60 to 60 seconds over a period of 630 seconds. To have the same magnification factor, the records were made from the same working distance for each eye, and then the data were processed. 12 images were saved as jpg files for each eye. The images were processed in the Mshot Imaging Analysis System program.

The substances to be researched were applied in the conjunctival sac by instillation, without touching the ocular surface, at moments T1 and T6. A drop of distilled water was administered 30 seconds after the start of the recording, and a drop of 1.5 mmol/ L adrenaline solution was administered at 330 seconds. Moments T1 and T6 were not analyzed. The vascular diameter measurement moments were: T0 (0 seconds), T1 (30 seconds), T2 (90 seconds), T3 (150 seconds), T4 (210 seconds), T5 (270 seconds), T6 (330 seconds), T7 (390 seconds), T8 (450 seconds), T9 (510 seconds), T10 (570 seconds) and T11 (630 seconds). Moments T1 and T6, when the substances to be investigated were applied, were not analyzed.

The parameters followed were variations in vascular caliber (vasodilation/ vasoconstriction), and the measurements were expressed in micrometers.

For each eye, respectively for each image of the chosen moment T0-T11, 3 measurements of the external diameter were performed at the same points for which the average was calculated. Subsequently, for each moment of each determination, the percentage variation of the diameter of the new blood vessels relative to the time T0 was calculated according to the following formula:

$$Drel=(\frac{Dx-D0}{D0})*100$$

where Drel represents the mean of the percentage variation of the blood vessel diameter from the moment T0, Dx represents the diameter in µm of the blood vessel at the measured moment, and D0 represents the diameter of the blood vessel in µm from the moment T0.

The positive values of Drel are represented by the increases in the diameter (vasodilation), while the negative values are the expression of the decrease of the vascular diameter (vasoconstriction).

The mean and the standard error were calculated for each batch and each moment of the determinations. Using the T-Student test, the variant for paired samples (2-tailed, 1 paired), the statistical significance of the difference between each moment and the T0 moment was calculated, comparing Drel with the value from the T0 moment. The results were considered statistically significant if p<0.05.

## Results

**Fig. 1:**
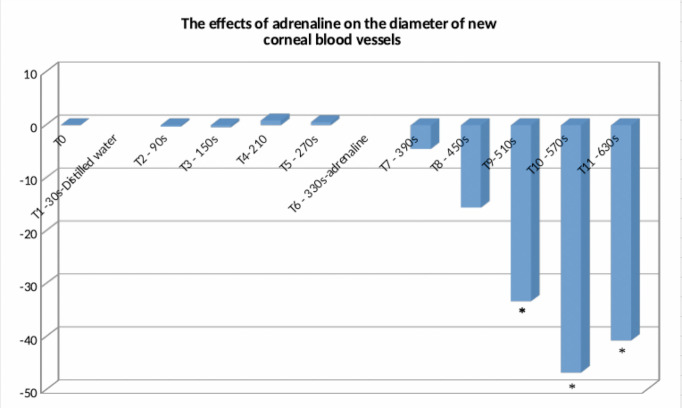
The evolution over time of the mean percentage change of vascular diameter after the administration of distilled water at the moment T1, respectively after the administration of adrenaline 1.5mmol/L, at the moment T6. The moments at which the determinations were performed are represented horizontally, the mean percentage variation of the vascular diameter is represented vertically. There were statistically significant changes for moments T9, T10, and T11 (* p<0.05)

After administration of distilled water at time T1, the mean percentage change in blood vessel diameter ± standard error was -0.28 ± 0.92 at time T2, -0.43 ± 0.87 at time T3, 0.89 ± 0.8 at time T4, and 0.57 ± 1.1 at time T5, the differences being statistically insignificant compared to time T0. After administration of adrenaline 1.5 mmol/ L at time T6, the mean percentage change in vascular diameter ± standard error was -4.5 ± 4.38 at time T7, -15.59 ± 7.82 at time T8, -33.32 ± 8.7 at time T9, -46.72 ± 9.72 at time T10, and -40.71 ± 8.88 at time T11, for the last 3 values the differences being statistically significant compared to time T0. At the T11 moment, the vascular diameter for 2 of the examined eyes decreased so much that the optical-electronic system used for recording no longer allowed its measurement. The results are presented in **Table 3** and **Fig. 1**.

**Tabel 3:** Evolution over time of the mean percentage change in the diameter of the corneal blood vessels after the administration of distilled water at time T1, respectively after the administration of adrenaline 1.5 mmol/ L at time T6

Image capture time (seconds)	Specimen	1	2	3	4	5	6	Mean	Std.Er.	p-value
	T0	0	0	0	0	0	0	0	0	0
	T1 -30s- Administration of distilled water									
	T2 - 90s	2.94	0	-1.33	1.66	-2.08	-2.86	-0.28	0.92	0.77
	T3 - 150s	2.94	0	-2.66	0	0	-2.86	-0.43	0.87	0.64
	T4-210	2.94	3.7	-1.33	0	0	0	0.89	0.8	0.32
	T5 - 270s	1.47	3.7	-2.66	1.66	2.08	-2.86	0.57	1.1	0.63
	T6 - 330s- Adrenaline administration									
	T7 - 390s	8.82	0	-10.66	-21.66	2.08	-5.55	-4.5	4.38	0.35
	T8 - 450s	8.82	-14.81	-9.33	-40	-2.08	-36.11	-15.59	7.82	0.10
	T9-510s	-14.7	-18.52	-9.33	-48.33	-56.25	-52.77	-33.32	8.7	0.01
	T10 -570s	-42.64	-22.22	-18.66	-61.66	-54.58	-80.55	-46.72	9.72	0.004
	T11 -630s	-41.17	-33.33	-23.33	-65	DNM	DNM	-40.71	8.88	0.019
Std.Er. = Standard error, DNM = non-measurable diameter										

## Discussions

**Fig. 2:**
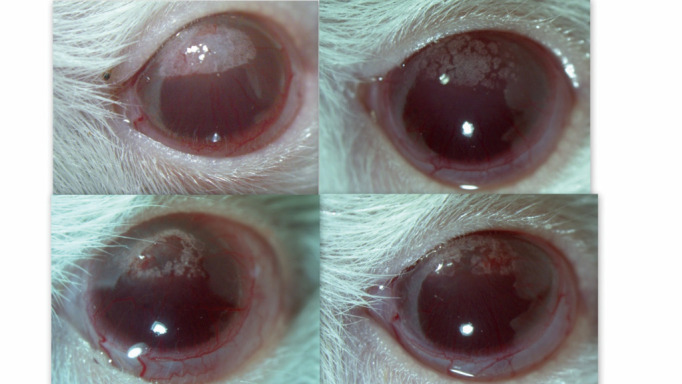
Images showing change transparency and corneal blood vessels

The model of corneal neovascularization is the result of research done to investigate sodium selenite-induced cataract in 15-day-old rat pups in which, for microscopic study of lens opacities, general anesthesia was performed with ketamine at a dose of 150 mg/ kg body weight and in which in vivo study of lens transparency changes was no longer possible due to the occurrence of changes in corneal transparency (**Fig. 2**). The determining factor in the production of corneal changes was further investigated, and the conclusion was that ketamine is responsible for these changes in corneal transparency, which is consistent with existing data in literature [**44**-**47**].

The results presented above showed that the administration of adrenaline produces a statistically significant decrease in the diameter of the blood vessels (vasoconstriction). In two of the eyes examined, the vasoconstriction was so intense that the measurement of the vascular diameter was no longer possible. The administration of distilled water did not produce statistically significant changes in vascular diameter. These allowed us to assume that there are α-adrenergic receptors at the level of the corneal blood vessels, whose stimulation classically produces vasoconstriction.

Following these results, we cannot exclude the existence of β-adrenergic receptors whose stimulation produces vasodilation. The vasoconstriction found above may be an algebraic sum between the vasoconstrictor effect produced by stimulating α-adrenergic receptors and the vasodilatory effect produced by stimulating β-adrenergic receptors if the vasodilatory effect is less intense than the vasoconstrictor effect.

## Conclusions

1. Administered in conjunctival instillations, adrenaline produces vasoconstriction in the corneal blood vessels.

2. In our experimental conditions, there were α-adrenergic receptors in the corneal blood vessels.

3. It is possible that there are also β-adrenergic receptors in the corneal blood vessels, but whose stimulation produces lower intensity vasodilation, masked by the vasoconstrictor effect produced by the stimulation of α-adrenergic receptors.

**Conflict of Interest**

Authors state no conflict of interest.

**Acknowledgements**

None.

**Sources of Funding**

None.

**Disclosures**

None.
